# Regulatory Mechanism of ABA and ABI3 on Vegetative Development in the Moss *Physcomitrella patens*

**DOI:** 10.3390/ijms19092728

**Published:** 2018-09-12

**Authors:** Mengkai Zhao, Qilong Li, Zhenhua Chen, Qiang Lv, Fang Bao, Xiaoqin Wang, Yikun He

**Affiliations:** 1 College of Life Science, Capital Normal University, Beijing 100048, China; zhaomk66@yeah.net (M.Z.); nardlee@163.com (Q.L.); 2150802083@cnu.edu.cn (Z.C.); 2132008021@cnu.edu.cn (Q.L.); 5838@cnu.edu.cn (F.B.); 2 Beijing Collaborative Innovation Center for Eco-Environmental Improvement with Forestry and Fruit trees, Beijing University of Agriculture, Beijing 102206, China

**Keywords:** ABA treatment, auxin-related genes, Physcomitrella patens, PpABI3, RNA-seq, vegetative development

## Abstract

The moss *Physcomitrella patens* is a model system for studying plant developmental processes. ABSCISIC ACID INSENSITIVE3 (ABI3), a transcription factor of the ABA signaling pathway, plays an important role in plant growth and development in vascular plant. To understand the regulatory mechanism of ABA and PpABI3 on vegetative development in *Physcomitrella patens*, we applied physiological, cellular, and RNA-seq analyses in wild type (WT) plants and ∆*abi3* mutants. During ABA treatment, the growth of gametophytes was inhibited to a lesser extent ∆*abi3* plants compared with WT plants. Microscopic observation indicated that the differentiation of caulonemata from chloronemata was accelerated in ∆*abi3* plants when compared with WT plants, with or without 10 μM of ABA treatment. Under normal conditions, auxin concentration in ∆*abi3* plants was markedly higher than that in WT plants. The auxin induced later differentiation of caulonemata from chloronemata, and the phenotype of ∆*abi3* plants was similar to that of WT plants treated with exogenous indole-3-acetic acid (IAA). RNA-seq analysis showed that the PpABI3-regulated genes overlapped with genes regulated by the ABA treatment, and about 78% of auxin-related genes regulated by the ABA treatment overlapped with those regulated by PpABI3. These results suggested that ABA affected vegetative development partly through PpABI3 regulation in *P. patens*; PpABI3 is a negative regulator of vegetative development in *P. patens*, and the vegetative development regulation by ABA and PpABI3 might occur by regulating the expression of auxin-related genes. PpABI3 might be associated with cross-talk between ABA and auxin in *P. patens*.

## 1. Introduction

Abscisic acid (ABA) is a well-known phytohormone that regulates seed dormancy and germination, stomatal closure, and response to environmental stress. In addition, ABA regulates numerous aspects of plant growth and development. Previous studies have identified the importance of ABA in the fruit ripening of strawberry [1]. Normal levels of endogenous ABA are required to maintain shoot development and leaf expansion in tomato [2]. The role of ABA in regulating the floral transition has also been reported. Increase in ABA can induce overexpression of the ABA-insensitive genes ABI4 and ABI5, which may delay floral transition, suggesting that ABA inhibits flowering [3,4]. Whereas the increased content of ABA is a response to drought, ABA positively influences flowering [5]. In *Arabidopsis thaliana*, hypocotyl elongation is suppressed by ABA with dephosphorylation of H^+^-ATPase in etiolated seedlings [6]. Several ABA signaling components have been indicated to regulate root growth and root system architecture. The ABA receptor PYL9 (PYRABACTIN RESISTANCE-LIKE PROTEIN 9), together with PYL8, is responsible for the recovery of lateral roots from ABA inhibition [7]. The gene products ABI1, ABI2, ABI3, ABI4, ABI5, and ABI8, important regulators of the ABA signal transduction pathway, are involved in regulating root development [8,9,10,11].

ABSCISIC ACID INSENSITIVE 3 (ABI3), a positive regulator of the ABA signaling pathway, plays an important role in seed development and dormancy. During seed development, ABA is known to control embryo maturation and desiccation tolerance through ABI3 regulating the expression of several seed storage proteins, lack of ABI3 reduces seed dormancy [12]. ABI3 also has broader functions, such as plastid development, bud dormancy, and somatic embryogenesis. When Arabidopsis seedlings are transferred from light to darkness, *abi3* mutants contain typical etioplasts, WT leaves contain arrested plastids, and the leaves of transgenic plants *EN35S::ABI3* (overexpression of ABI3) contain chloroplasts. These results suggest that AtABI3 is responsible for plastid differentiation in vegetative tissues [13]. In poplars (*Populus trichocarpa*), PtABI3 is expressed in the embryonic leaves of the bud during bud set in short-day conditions and regulates the relative growth rate and differentiation of embryonic leaves inside the bud [14]. The overexpression of BABY BOOM (BBM), a transcription factor related to plant cell totipotency, induces Arabidopsis somatic embryo formation; moreover, AtABI3 quantitatively regulates BBM-mediated somatic embryogenesis [15]. In addition, the expression of the ABI3 gene is induced by auxin in the root, and ABI3 has been shown to promote lateral root formation and inhibit germination in response to auxin [9,16,17].

ABI3 is conserved in the evolution of angiosperm plants and bryophytes. As a pioneer plant from aquatic to terrestrial, the moss *Physcomitrella patens* was the first bryophyte to have its entire genome sequenced [18]. Three homologous genes, *PpABI3A/B/C*, are present in *P. patens*. PpABI3A can partially restore the phenotype of yellow seed skin of the mutant *abi3-6* in *A. thaliana* but cannot fully restore ABA sensitivity in the process of seed germination [19]. In *P. patens*, deletion mutants *abi3a/b/c* (∆*abi3*) are not able to recover from desiccation stress, suggesting that PpABI3 is responsible for the survivability of *P. patens* vegetative tissues upon desiccation [20]. PpABI3 is also involved in regulating cold acclimation and freezing tolerance in *P. patens* [21]. However, little is known about the mechanism by which PpABI3 regulates the growth and development of in *P. patens*.

In this study, we employed physiological, cellular, and RNA-seq analyses to examine the changes in auxin concentration, cell shape, and the global gene expression in WT plants and ∆*abi3* plants under ABA treatment. Our study may provide important insight into understanding the mechanism of ABA and PpABI3A on vegetative development in *P. patens*.

## 2. Results

### 2.1. ∆abi3 Plants Were Insensitivity to ABA Treatment When Compared with WT Plants

**Figure 1:**
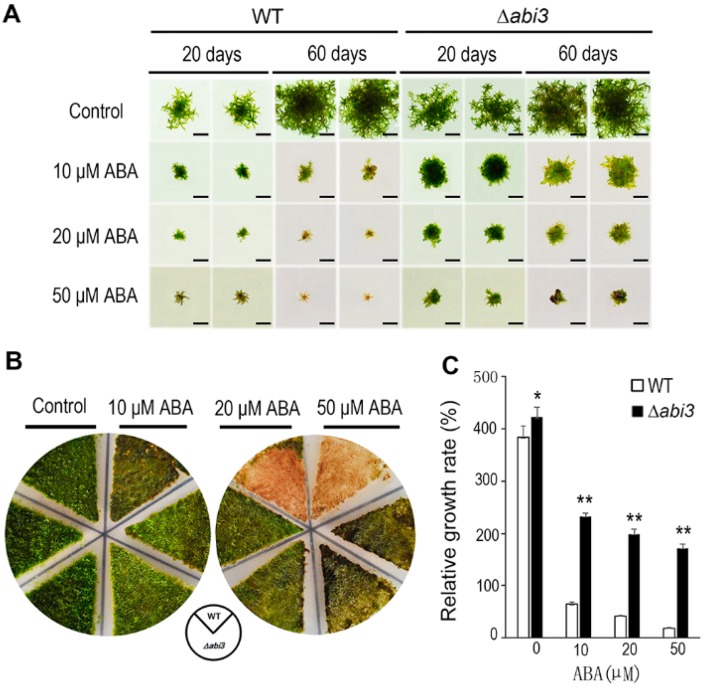
Phenotype changes in WT and ∆*abi3* plants during ABA treatment. (**A**) A tip of leaf-like structure of gametophytes of WT and ∆*abi3* plants were cultured on BCD medium containing 0, 10, 20, 50 μM ABA for 20 days and 60 days. Bar = 1 cm. (**B**) 7-day-old protonemata of WT and ∆*abi3* plants were cultured on BCD medium containing 0, 10, 20, 50 μM ABA for 14 days. (**C**) Relative growth rate of gametophytes of WT and ∆*abi3* plants were cultured on BCD medium with 0, 10, 20, 50 μM ABA for 20 days and 60 days. Data are means of three independent replicates ± standard error (SE). * indicates a significant difference compared to the control (0.01 < *p* < 0.05); ** indicates an extremely significant difference compared to the control (*p* < 0.01). The control is the relative growth rate of WT.

To investigate the function of ABI3 in *P. patens* during growth and development, WT and ∆*abi3* plants were cultured on BCD medium containing 0, 10, 20, and 50 μM of ABA. Although the growth of gametophores was inhibited in both WT and ∆*abi3* plants during ABA treatment, the growth inhibition of ∆*abi3* plants was much weaker than that of WT plants under ABA treatment conditions (Figure 1A). As shown in Figure 1B, in 7-day-old protonemata treated with 10 μM of ABA for 14 days, the tissues of WT with 10 μM of ABA treatment became browner than those without ABA treatment, and the tissues of WT with 10 μM of ABA treatment were browner than those of ∆*abi3* mutants. When 7-day-old protonemata were treated for 14 days with 20 and 50 μM of ABA, the tissues of WT with 20 and 50 μM of ABA treatment became much browner than those without ABA treatment, and the tissues of WT with 20 and 50 μM of ABA treatment were much browner than those of ∆*abi3* mutants.

When the plants with 10, 20, and 50 μM of ABA treatment were compared to the plants without ABA treatment, relative growth rates of gametophores in WT plants were 16.7%, 10.9%, and 4.9%, respectively; and relative growth rates of gametophores in ∆*abi3* plants were 59.6%, 51.2%, and 44.0% respectively (Figure 1C). Obviously, the growth of *P. patens* was inhibited by ABA, the sensitivity to ABA treatment was reduced in ∆*abi3* plants, compared with WT, suggesting that the function of PpABI3 is involved in *P. patens* growth through regulation of the ABA signaling pathway.

### 2.2. Caulonema Formation Was Accelerated and Chloronema Branching Was Reduced in ∆abi3 Plants, However, ABA Inhibited This Phenotype

**Figure 2:**
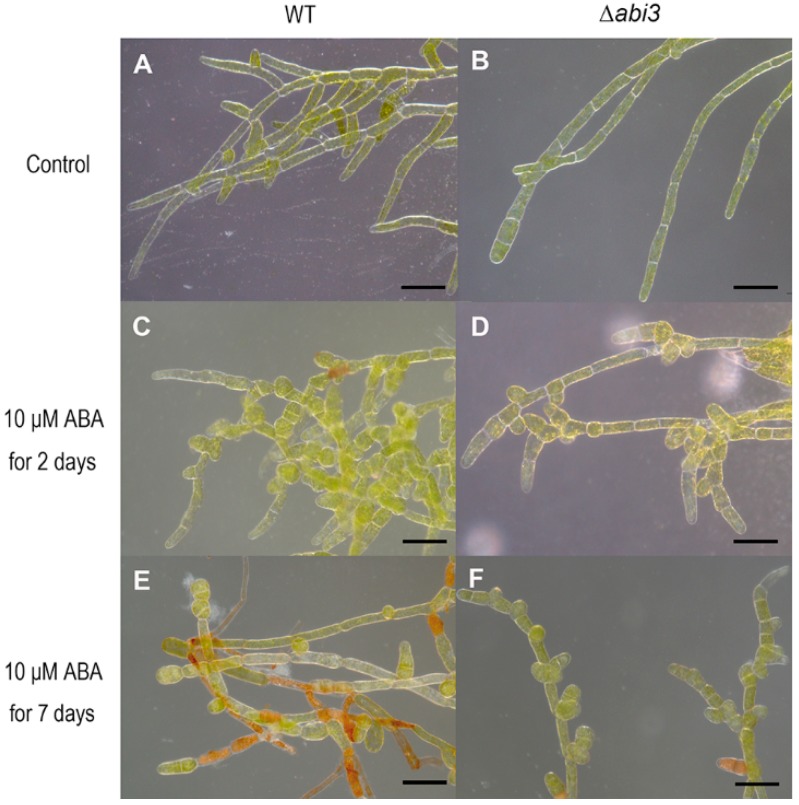
Stereoscopic images of WT and ∆*abi3* plants during ABA treatment. (**A**,**B**) 7-day-old protonemata of WT and ∆*abi3* plants grown under normal condition. (**C**,**D**) 7-day-old protonemata were cultured on BCD medium with 10 μM ABA for 2 days. (**E**,**F**) 7-day-old protonemata were cultured on BCD medium with 10 μM ABA for 7 days. Bar = 100 μm.

To characterize the cell growth and development of *P. patens* with or without ABA treatment, the cell shape of WT and ∆*abi3* plants was observed using a stereoscopic microscope. The results showed that ∆*abi3* mutants exhibited slight elongation of cells, caulonema formation was accelerated and chloronema branching was reduced in the absence of ABA when compared with that of WT plants (Figure 2A,B). We also found that ABA caused cell swelling and brood cells formation in both WT and ∆*abi3* plants under a 10 μM of ABA treatment (Figure 2C–F). When exposed to exogenous ABA or external stress conditions, cells of WT protonemata were prone to differentiate into spherical brood cells [22]. Moreover, the effect in WT plants was much more obvious than that in the ∆*abi3* plants (Figure 2C–F). These observations suggested that ABA is responsible for a change in cell shape, producing thicker and less elongated cells.

**Figure 3:**
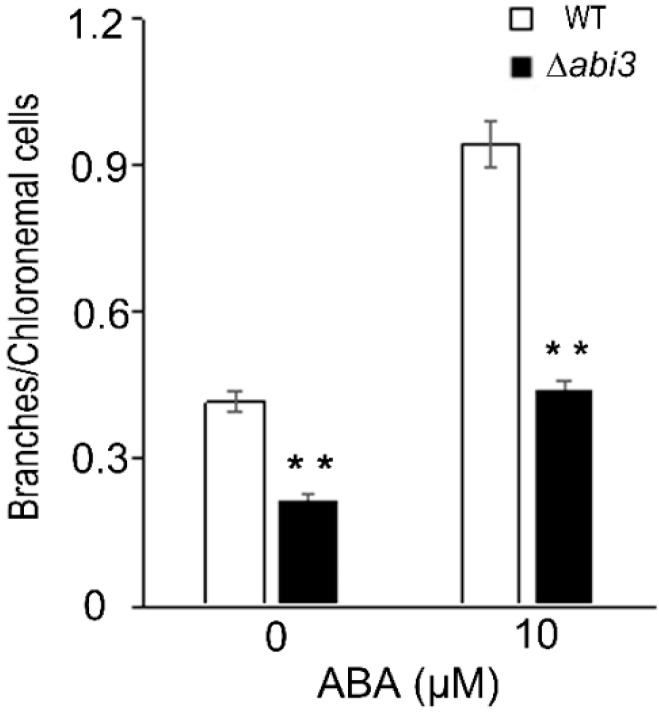
The branch changes in WT and ∆*abi3* plants during ABA treatment. The branches of per chloronemal cell in 7-day-old protonemata of WT and ∆*abi3* plants were cultured on BCD medium containing 0 and 10 μM ABA for 2 days. Data are means of thirty independent replicates ± SE. ** indicates an extremely significant difference compared to the control (*p* < 0.01). The control is the branches of per chloronemal cell of WT.

Protonemata are composed of chloronemata and caulonemata. Chloronemata form upon germination, and caulonemata later differentiate from chloronemata. Previous studies have shown that the increase of chloronemal branching is negatively correlated with caulonema formation [23]. Under a 10 μM of ABA treatment for 2 days, the number of branches per chloronemal cell was enhanced in both WT and ∆*abi3* plants (Figure 3), suggesting that caulonema formation was inhibited by ABA. Compared with WT plants, the number of branches per chloronemal cell was reduced in ∆*abi3* plants (Figure 3), indicating that caulonema formation was accelerated in ∆*abi3* plants with 10 μM of ABA treatment or without ABA.

### 2.3. Gametophyte Growth Was Faster and Auxin Concentration Was Higher in ∆abi3 Plants Than Those of WT Plants

**Figure 4:**
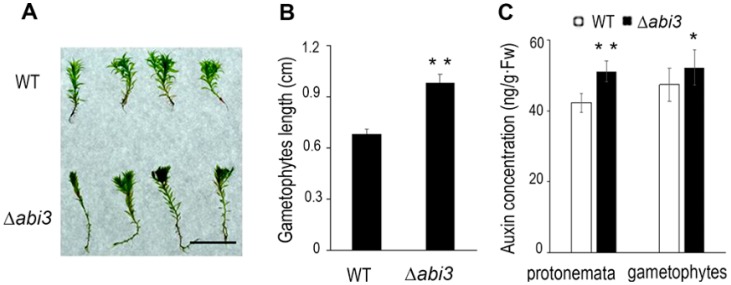
Gametophyte length and auxin concentration changes of WT and ∆*abi3* plants. (**A**) 30-day-gametophytes of WT and ∆*abi3* plants. Bar = 1 cm. (**B**) The height measurement of 30-day-gametophytes of WT and ∆*abi3* plants. Data are means of sixty independent replicates ± SEM. (**C**) Auxin concentration in the tissues of 7-day-old protonemata and 30-day-gametophytes of WT and ∆*abi3* grown under normal condition. Data are means of five independent replicates ± SE. * indicates a significant difference compared to the control (0.01 < *p* < 0.05); ** indicates an extremely significant difference compared to the control (*p* < 0.01). The control is the gametophytes length or auxin concentration of WT.

To further compare the difference in growth and development of WT and ∆*abi3* plants, the percentage of the lengths in a stem-like structure of gametophytes and the auxin concentration was measured. Under normal growth conditions, the lengths of a stem-like structure of 30-day-old gametophytes in ∆*abi3* plants were about 1.5 times longer than those of WT plants (Figure 4A,B). Some research has shown that the lengths of a stem-like structure of gametophytes are positively correlated with auxin at physiological concentrations [24], whereas the number of branches per chloronemal cell (Figure 3) is negatively correlated with auxin at physiological concentrations [23]. As Figure 4C shows, whether protonemata or gametophytes, auxin concentrations in ∆*abi3* plants were significantly higher than those of WT plants under normal growth conditions. These results suggest that auxin concentration might be related to the difference in growth and development of WT and ∆*abi3* plants.

### 2.4. IAA Induced Caulonemata Formation and Inhibited the Formation of Chloronema Branches in WT Plants

**Figure 5:**
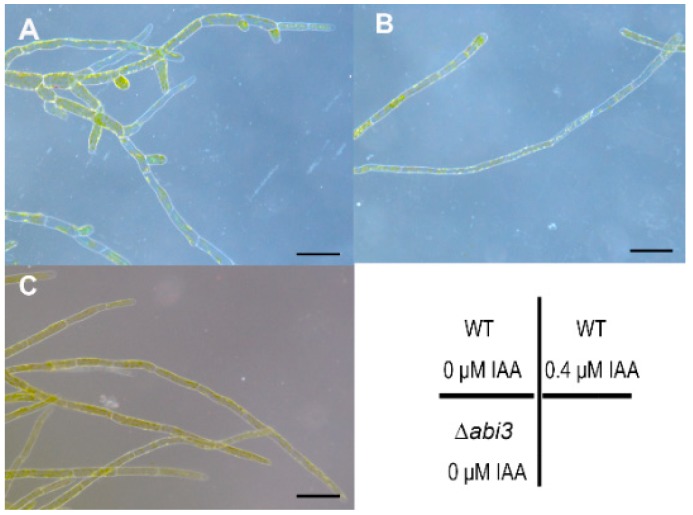
Stereoscopic images of WT plants during IAA treatment. (**A**,**B**) 7-day-protonemata of WT were cultured on BCD medium with 0 and 0.4 μM IAA for 5 days. (**C**) 12-day-protonemata of ∆*abi3* grown under normal conditions. Bar = 100 μm.

To study the effect of auxin on growth and development in *P. patens*, 7-day-old protonemata of WT were cultured on BCD medium with 0 and 0.4 μM of IAA for 5 days, and protonemata of ∆*abi3* plants were cultured on normal BCD medium for 12 days. The cell shape was then observed using stereoscopic microscopy. The number of chloronema branches in WT plants without IAA treatment (Figure 5A) were much higher than that of WT plants with 0.4 μM of IAA treatment (Figure 5B) and 12-day-old protonemata of ∆*abi3* plants (Figure 5C), suggesting that auxin could induce the later differentiation of caulonemata from chloronemata, and that the phenotype of ∆*abi3* plants is similar to WT plant treatment with exogenous IAA. Together, ABI3 could mediate the growth and development of *P. patens*, and its function as a negative regulator affects growth and development.

### 2.5. Role of ABA and ABI3 in Global Gene Expression

**Figure 6:**
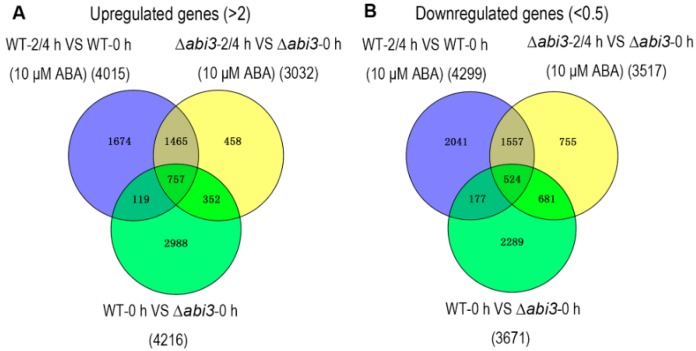
Differential expression analysis of genes of WT and ∆*abi3* plants with or without ABA treatment. The overlaps of upregulated genes (**A**) and downregulated genes (**B**) between WT plants during ABA treatment (purple), ∆*abi3* plants during ABA treatment (yellow) and WT plants compared with ∆*abi3* plants under normal growth conditions (green).

To better understand the molecular mechanism of ABA and ABI3 on the growth and development of *P. patens*, RNA-seq analysis was performed in six groups of WT protonemata and ∆*abi3* protonemata treated with 10 μM of ABA for 0 h, 2 h, and 4 h. High-throughput Illumina sequencing technology was used to profile the *P. patens* mRNA transcripts, and 32,926 genes were identified. The differentially expressed genes (DEGs) were determined by a standard with fold change ≥2 and false discovery rate (FDR) test *p* < 0.05 among the WT, ∆*abi3*, and their ABA treatment groups. As Figure 6 shows, the Venn diagrams of DEGs indicated that 4015 genes were upregulated and 4299 genes were downregulated in WT plants during ABA treatment, and 3032 genes were upregulated and 3517 genes were downregulated in ∆*abi3* plants during ABA treatment. There is an overlap of 2222 upregulated genes and 2081 downregulated genes between WT and ∆*abi3* plants during ABA treatment, suggesting that the expression of these genes was regulated by an ABA signaling pathway independent of ABI3. A total of 7887 genes were differentially expressed, with 4216 upregulated genes and 3671 downregulated genes when WT plants were compared with ∆*abi3* plant under normal growth conditions. Among them, 2988 (70.9%) genes were upregulated in WT plants but not induced by ABA signaling, and 2289 (62.4%) genes were downregulated in WT plants but not inhibited by ABA signaling. There is an overlap of 757 upregulated genes and 524 downregulated genes among the DEGs of WT plants during ABA treatment, ∆*abi3* plants during ABA treatment, and WT plants compared with ∆*abi3* plants under normal growth conditions, indicating that the expression of these genes was regulated by both ABI3 and the ABA signaling pathway independent of ABI3.

### 2.6. Role of ABA and ABI3 in Growth and Development Gene Expression

**Figure 7:**
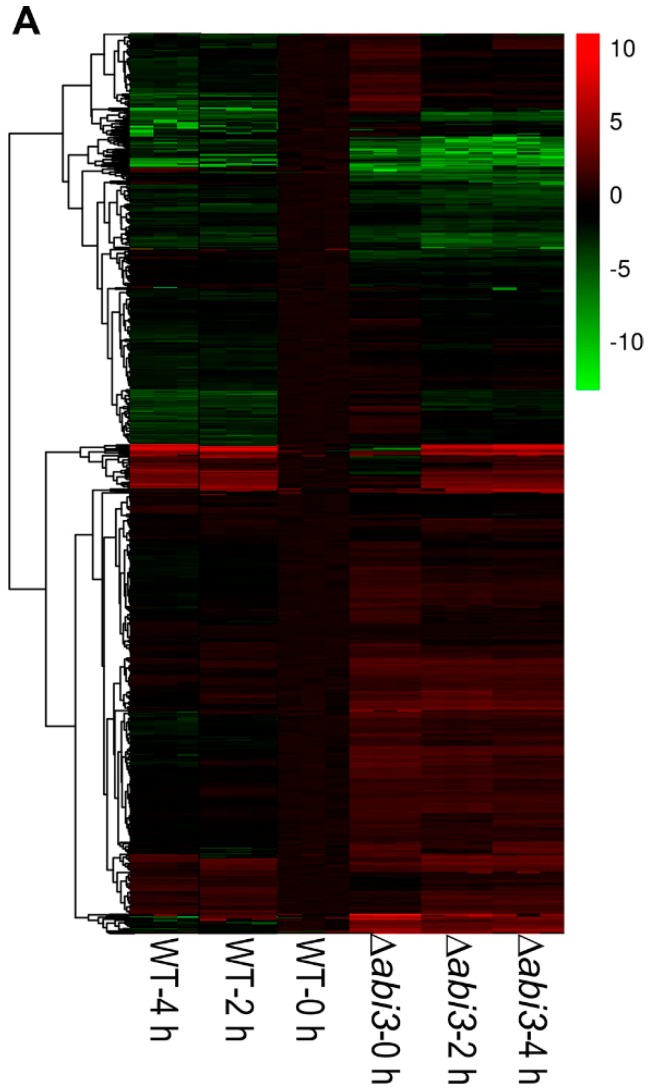

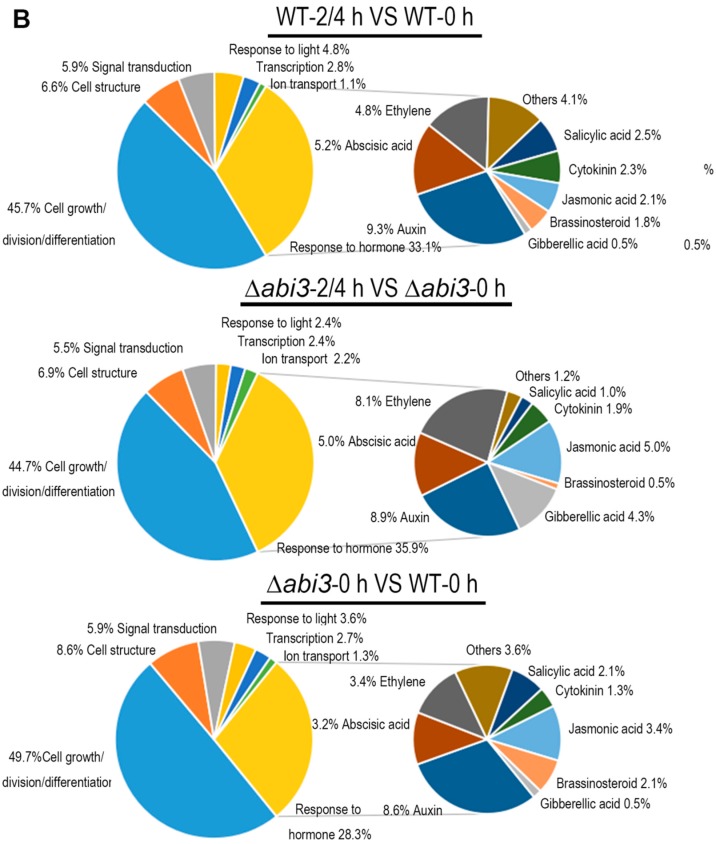
Differential expression analysis of genes involved in growth and development when WT and ∆*abi3* plants with or without ABA treatment. (**A**) Hierarchical clustering for the expression pattern of the differentially expressed genes involved in growth and development (WT 0 h compared with WT 2 h, WT 4 h, ∆*abi3* 0 h, ∆*abi3* 2 h, and ∆*abi3* 4 h). The colour represents the expression level of the gene. The range of induction and repression is shown from dark to red and green. (**B**) Annotated functional categorization of the differentially expressed genes involved in growth and development. In the subcategories, the group of others means the genes response to several hormones.

The gene ontology (GO) function annotation was performed to further understand the biological process of DEGs, and there were 746 genes related to growth and development (Tables S1–S7). Figure 7A shows a cluster analysis of the 746 genes of growth and development from the RNA-seq data for WT plants versus ∆*abi3* plants, WT plants versus WT plants of ABA treatment, and ∆*abi3* plants versus ∆*abi3* plants of ABA treatment. During ABA treatment, the expression of 746 genes showed significant changes, and this suggested that growth and development was regulated in *P. patens*. When ∆*abi3* plants were compared with WT plants, many genes also showed significant changes in ∆*abi3* plants, indicating that ABI3 could regulate the expression of the genes involved in growth and development; this might explain the difference in phenotype of ∆*abi3* plants compared with WT plants under normal culture conditions.

The 746 genes related to growth and development were categorized according GO function annotation, based on the European Union (EU) *A. thaliana* genome project [25]. Regarding putative function, the genes could be sorted into seven categories (Tables S1–S7; Figure 7B), the largest of which was cell growth/division/differentiation. The second-largest category was response to hormone and the next-largest category was cell structure. The other categories were signal transduction, response to light, transcription, and ion transport. The genes in the group of response to hormone were mainly those involved in auxin, ABA, and ethylene. As expected, the largest subcategory was response to auxin in WT and ∆*abi3* plants during ABA treatment and ∆*abi3* plants compared with WT plants under normal growth conditions. Thirty-two important auxin-related genes (Table 1) were chosen for further analysis.

### 2.7. Role of ABA and ABI3 in Auxin-Related Gene Expression

**Figure 8:**
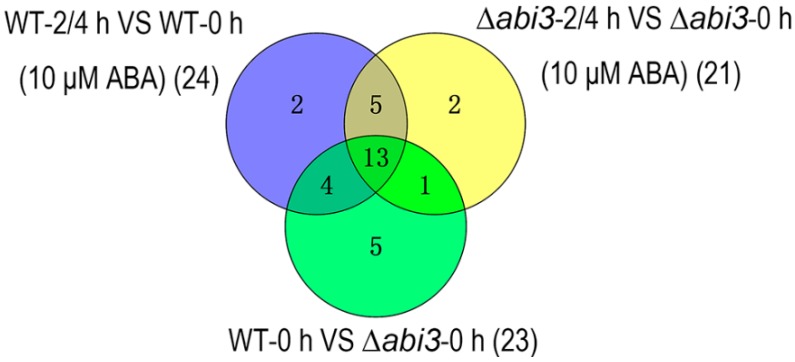
The overlap of differentially expressed auxin-related genes between WT plants during ABA treatment (purple), ∆*abi3* plants during ABA treatment (yellow), and ∆*abi3* plants compared with WT plants with or without ABA treatment (green).

Among the 32 important auxin-related genes (Figure 8; Table 1), 24 auxin-related genes were identified during ABA treatment of WT plants, 21 auxin-related genes were identified during ABA treatment of ∆*abi3* plants, and 23 auxin-related genes were identified when ∆*abi3* plants compared with WT plants. There were 13 auxin-related genes overlapped among the 32 genes of WT plants during ABA treatment, of ∆*abi3* plants during ABA treatment, and of ∆*abi3* plants compared with WT plants. A total of 23 auxin-related genes were differentially expressed with 14 upregulated genes and nine downregulated genes when ∆*abi3* plants were compared with WT plants, suggesting that ABI3 could mediate the expression of auxin-related genes. Twenty-three of 24 auxin-related genes were downregulated during ABA treatment of WT plants, and 20 of 21 auxin-related genes were downregulated during ABA treatment of ∆*abi3* plants, indicating that ABA can inhibit the expression of auxin-related genes.

The YUC genes were first identified as auxin biosynthesis enzymes, and are rate-limiting enzymes in the auxin synthesis pathway [26]. One YUC gene (Pp3c13_21970) was downregulated during ABA treatment of WT plants and ∆*abi3* plants; one YUC gene (Pp3c1_11500) was upregulated during ABA treatment of ∆*abi3* plants; three YUC genes (Pp3c11_11790, Pp3c13_21970, and Pp3c1_11500) were upregulated when ∆*abi3* plants were compared with WT plants with or without ABA treatment.

Auxin is mainly transported by three proteins: inflow carrier (AUX1/LAX), outflow carrier (PIN), and ATP-binding transporter (ABCB/MDR/PGP) [27,28,29,30]. Four AUX1 homologous genes (Pp3c12_5490, Pp3c15_640, Pp3c9_620, and Pp3c6_27190), three PIN homologous genes (Pp3c14_8850, Pp3c23_10200, and Pp3c24_2970), and two ABCB homologous gene (Pp3c6_7260 and Pp3c12_11230) were downregulated and one AUX1 homologous gene (Pp3c9_3590) was upregulated during ABA treatment of WT plants and ∆*abi3* plants. In addition, two AUX1 homologous genes (Pp3c22_23050 and Pp3c3_9870) were downregulated during ABA treatment of WT plants. When ∆*abi3* plants were compared with WT plants with or without ABA treatment, one PIN gene (Pp3c14_8850) and three ABCB genes (Pp3c17_16450, Pp3c6_7260, and Pp3c12_11230) were upregulated; seven AUX1 homologous genes (Pp3c12_5490, Pp3c15_640, Pp3c19_12670, Pp3c22_23050, Pp3c3_9870, Pp3c9_620, and Pp3c9_3590) and two PIN homologous genes (Pp3c23_10200 and Pp3c24_2970) were downregulated.

AUX/IAA family genes and auxin response factors (ARFs) are important components of the auxin signaling pathway [31,32,33]. Seven ARFs (Pp3c4_13010, Pp3c5_9420, Pp3c1_14480, Pp3c9_21330, Pp3c13_4720, Pp3c14_16990, and Pp3c15_9710) were downregulated during ABA treatment of WT plants and ∆*abi3* plants. One AUX/IAA gene (Pp3c24_6610) and three ARFs (Pp3c4_12970, Pp3c9_17560, and Pp3c15_21880) were downregulated during ABA treatment of WT plants. Three ARF genes (Pp3c2_25890, Pp3c6_21370, and Pp3c6_26890) were downregulated during ABA treatment of ∆*abi3* plants. Two AUX/IAA genes (Pp3c2_25890 and Pp3c8_14720) and five ARFs genes (Pp3c4_13010, Pp3c5_9420, Pp3c1_14480, Pp3c2_25890, and Pp3c4_12970) were upregulated when ∆*abi3* plants were compared with WT plants with or without ABA treatment. These results suggest that the expression of auxin-related genes is regulated by ABA and ABI3, which affects the growth and development of the moss *P. patens*.

### 2.8. qRT-PCR Analysis of Auxin-Related Genes

**Figure 9:**
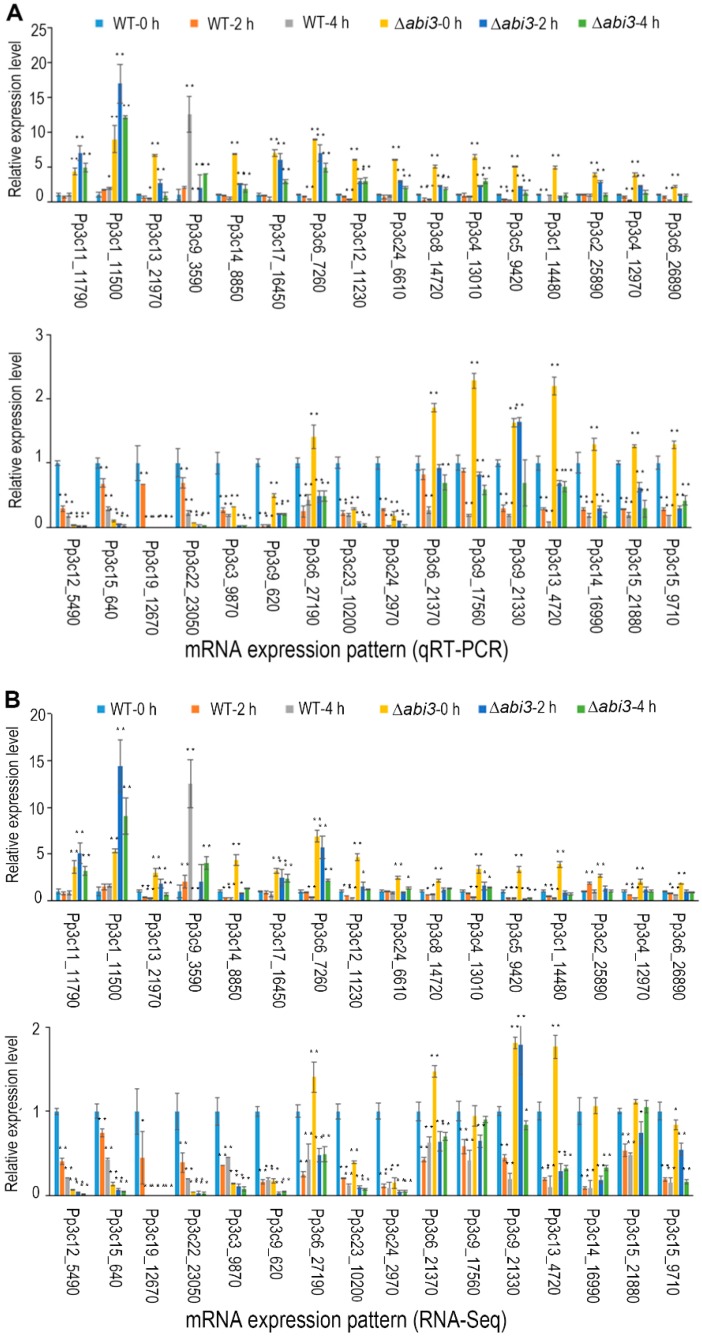
Differential expression analysis of auxin-related genes in WT and ∆*abi3* plants with or without ABA treatment. (**A**) Gene expression analysis by qRT-PCR. (**B**) Gene expression analysis by RNA-Seq. Data are the means of three independent replicates ± SE. * indicates a significant difference compared to the control (0.01 < *p* < 0.05); ** indicates an extremely significant difference compared to the control (*p* < 0.01). The control is the gene expression level of WT-0 h.

To confirm the expression profile of auxin-related genes in WT plants and ∆*abi3* plants as well as the same during ABA treatment, we performed real-time quantitative RT-PCR. We analyzed the expression of 32 important auxin-related genes that had been identified as DEGs. The results showed that the expression pattern of 32 auxin-related genes based on qRT-PCR (Figure 9A) was similar to that of RNA-seq data (Figure 9B).

## 3. Discussion

Phytohormones and plant growth regulators are important molecules required for various biological processes in plants. ABA is one such phytohormone that regulates various aspects of plant development, including seed dormancy and germination, fruit development, and root development [1,7,8,9,10,11]. ABI3, an important transcription factor in the ABA signaling pathway, plays a critical role in seed development and dormancy, plastid development, bud dormancy, and somatic embryogenesis [13,14,15]. Moreover, ABI3 is involved in auxin and ABA cross-talk during seed germination and lateral root emergence [9,16,17]. In this study, we reveal the regulation mechanism of ABA and ABI3 on vegetative development in the moss *P. patens*.

### 3.1. ABA and ABI3 Regulate Vegetative Development in P. patens

ABA biosynthesis and signaling response have been well studied during vegetative development in *A. thaliana*. The *AtABA1* gene encodes an enzyme involved in the ABA biosynthesis pathway. The deletion mutation of the *AtABA1* gene caused a reduction in the size of the leaves, inflorescences, and flowers. The fresh and dry weights of rosettes of *aba1* mutants were lower than those of WT plants. Low concentrations (50 nM) of exogenous ABA can increase the fresh weight of *aba1* mutants and WT plants, indicating that ABA acts as a growth promoter during vegetative development. In addition, the abnormal shape and internal structure of the leaves in *aba1* mutants suggest that ABA is responsible for organogenesis [34]. PYRABACTIN RESISTANCE 1 (PYR1)/PYR1-LIKE (PYL), clade A protein phosphatase type 2Cs (PP2C), and sucrose nonfermenting 1–related protein kinase 2 (SnRK2) are key players in the regulation of ABA signaling [35]. In the *snrk2.2/2.3/2.6* triple mutant of *A. thaliana*, early seedling development and growth were insensitive to ABA. Although high concentrations (50 μM) of exogenous ABA suppressed shoot growth both in *snrk2.2/2.3/2.6* triple mutants and WT plants, the shoot fresh weight of *snrk2.2/2.3/2.6* was much higher than that of WT plants [36]. High concentrations of ABA inhibit seedling growth of WT plants, whereas certain ABA-insensitive mutants are resistant to the inhibition of vegetative growth. An *A. thaliana* sextuple mutant *pyr1 pyl1 pyl2 pyl4 pyl5 pyl8*, impaired in six PYR/PYL receptors, was able to grow even on 100 mM of ABA. Both *pyr1 pyl1 pyl2 pyl4 pyl5 pyl8* and *snrk2.2/2.3/2.6* were notably resistant to ABA-mediated inhibition of growth [37]. AtABI1 (ABA-insensitive 1), a member of the PP2C subfamily A, functions as a negative regulator of ABA signaling pathways and is involved in hypocotyl elongation. Application of the phytohormone ABA (4.2 μM) to etiolated seedlings suppressed hypocotyl elongation within 30 min, but an ABA-insensitive mutant, *abi1-1*, did not show ABA inhibition of hypocotyl elongation [6].

In the moss *P. patens*, PpABI1 (PP2C) has also been reported as a negative regulator in the ABA signaling pathway. There are two members of ABI1 in *P. patens*. Although the protonemata of *ppabi1a* single mutants and *ppabi1b* single mutants exhibited normal growth and development in the absence of exogenous ABA, the *ppabi1a/b* double mutant grew very slowly compared with the WT or both the single mutants. Also, protonemata of the *ppabi1a/b* mutants developed dwarf gametophytes. PpABI1A/B is essential for proper growth, mainly for elongation of both protonemata and gametophytes under nonstress conditions [38]. Therefore, the elements in the ABA signaling pathway could regulate the growth and development of plants, and they are conserved between *A. thaliana* and *P. patens*. As another important transcription factor in the ABA signaling pathway, ABI3, functions as a positive regulator involved in vegetative desiccation stress in *P. patens* [20]. In addition, PpABI3 is responsible for growth and development in *P. patens*. The gametophytes of ∆*abi3* triple mutants grew faster than those of WT plants, and the ∆*abi3* mutant exhibits slight elongation of cells under normal conditions. Caulonema formation was accelerated and chloronema branching was reduced in ∆*abi3* plants (Figure 1, Figure 2 and Figure 5). Application of exogenous ABA inhibited the growth of gametophytes in both WT plants and ∆*abi3* plants, whereas ∆*abi3* plants were less sensitive to ABA compared with WT plants (Figure 1). Application of exogenous ABA resulted in protonemata browning, and the WT plants with ABA treatment were much browner than the ∆*abi3* plants (Figure 1). Protonema cells were prone to differentiate into spherical brood cells under external stress conditions [22]. ABA also caused brood cells formation and cell swelling in both WT and ∆*abi3* plants, and the effect in WT plants was much more obvious than that in ∆*abi3* plants (Figure 2). Caulonema formation was inhibited by ABA. Compared with WT plants, caulonema formation was accelerated in ∆*abi3* plants whether in the absence of ABA or ABA treatment (Figure 2). The gene expression analysis indicated that more than 33% of DEGs were mediated by ABI3 but not by ABA signaling, and about 8% of DEGs were regulated by both ABI3 and the ABA signaling pathway independent of ABI3 (Figure 6). Taken together, the results suggest that ABA suppresses the vegetative development of the moss *P. patens*; PpABI3 is a negative regulator of the vegetative development in *P. patens*; ABA regulates vegetative development partly through PpABI3, and other regulators might also be involved in regulating vegetative development in *P. patens*.

### 3.2. Regulatory Mechanism of ABA and ABI3 on Vegetative Development

The caulonema formation and gametophytes growth in ∆*abi3* plants were faster than those in WT plants (Figure 1, Figure 2 and Figure 4), and this is an auxin-increased phenotype. Previous studies have shown that exogenous auxins promote the differentiation of *P. patens* from chloronema to caulonema and inhibit the formation of chloronema branches [39,40]. As expected, auxin concentrations of protonemata and gametophytes in ∆*abi3* plants were significantly higher than those of WT plants under normal growth conditions (Figure 4). Exogenous IAA induced later differentiation of caulonemata from chloronemata in WT plants; this phenotype is similar to ∆*abi3* plants without IAA treatment (Figure 5). Therefore, auxin might be related to the difference in the growth and development of WT and ∆*abi3* plants with or without ABA treatment.

The molecular regulatory mechanism of ABA and ABI3 on vegetative development was studied using RNA-seq analysis, and the genes related to the synthesis, transport, and signaling pathways of auxin were identified (Table 1, Figure 9). Auxin plays a crucial role in various aspects of plant development, and auxin positively regulates plant development by the mediation of cell division, elongation, and differentiation. Many studies have shown that ABA interacts with auxin to regulate plant development. The expression of auxin reporter ProDR5:GUS was greatly reduced in roots and leaves of Arabidopsis under ABA treatment, suggesting auxin concentration or response is regulated by ABA [41]. In rice, ABA promoted auxin biosynthesis and polar auxin transport, leading to auxin accumulation in the root tip, and then regulate root hair elongation [42]. The mutant of several auxin-related genes affects ABA sensitivity or ABA activity in the process of plant development. The *arf2* mutant enhances ABA sensitivity during primary root growth, and ABA treatment alters auxin distribution in Arabidopsis primary root tips [43]. In contrast, *aux1* and *pin2* mutants are insensitive to ABA-dependent repression of embryonic axis elongation. ABA suppresses growth of the Arabidopsis embryonic axis elongation by combinatorial modulation of auxin transport facilitators AUX1 and PIN2 [44]. Compared with WT plants, the auxin signaling mutants *axr1*, *axr2/iaa7*, *slr/iaa14*, *iaa16*, and *axr3/iaa17* showed differential response to ABA treatment, suggesting that Aux/IAA-dependent auxin signaling affects ABA activity [45,46,47]. In this study, the auxin-related genes of YUC, AUX, PIN, ABCB, AUX/IAA, and ARF were downregulated in both WT plants and ∆*abi3* plants in *P. patens* during ABA treatment (Table 1; Figure 9), suggesting that ABA regulates the vegetative development of *P. patens* through cross-talk with auxin synthesis, transport, and signaling pathways.

Some ABA-INSENSITIVE factors are considered to be part of the interaction between auxin and ABA. ABI1, a key negative regulator of ABA signaling, exhibits decreased sensitivity to exogenous auxin in lateral root formation and is essential for auxin-modulated root development [48]. ABI3 is required for auxin-activated seed dormancy. The auxin response factors AUXIN RESPONSE FACTOR 10 and AUXIN RESPONSE FACTOR 16 are recruited by ABI3 to mediate the expression of *ABI3*, and then the network of auxin and ABA signaling is coordinated in the process of seed germination [17]. The decrease of auxin levels in roots leads to a reduction in lateral root formation and elongation. ABA reduces auxin transport in roots through ABI4 mediating the expression of PIN-FORMED 1, resulting in suppression of lateral root development. ABI5 also mediates the expression of PIN1 and root development [49]. Enhanced expression of ABI5 resulted in reduced accumulation of the PIN1 protein, thus leading to reduced auxin levels in roots and a reduction in the number and lengths of root apical meristem cells [50]. In our study, the auxin-related genes of YUC, AUX, AUX/IAA, and ARF were all upregulated, and most auxin-related genes of PIN and ABCB were downregulated in ∆*abi3* plants compared with WT plants with or without ABA treatment (Table 1, Figure 9), indicating that ABI3 regulates the vegetative development of *P. patens* by controlling the expression of auxin synthesis, transport, and signaling pathways, resulting in changes un auxin levels in the protonemata and gametophytes of *P. patens*.

In conclusion, ABA and PpABI3 could regulate the vegetative development of the moss *P. patens*. Application of exogenous ABA suppressed the vegetative growth in both WT plants and ∆*abi3* mutants. The growth rate of ∆*abi3* mutants was faster than that of WT plants with or without ABA treatment. Therefore, PpABI3 is a negative regulator of the vegetative development in *P. patens*. ABA regulated vegetative development partly through PpABI3, and other regulators might be also involved in regulating vegetative development in the moss *P. patens*. Another, auxin concentrations of protonemata and gametophytes in ∆*abi3* mutants was significantly higher than that of WT plants. Exogenous IAA promoted the differentiation of WT plants from chloronema to caulonema and this phenotype was similar to ∆*abi3* mutants without IAA treatment. Auxin-related genes were identified in WT plants and ∆*abi3* mutants with or without ABA treatment, indicating that ABA and PpABI3 regulated vegetative development of *P. patens* through controlling the expression auxin synthesis, transport, and signaling pathways and PpABI3 might be associated with cross-talk between ABA and auxin in the moss *P. patens*.

## 4. Materials and Methods

### 4.1. Plant Materials

Plant tissues of WT *P. patens* and ∆*abi3* mutant (Khandelwal et al. 2010) were grown on BCD (gametophytes) or BCDA (protonemata) media at 25 °C under 16 h light (30 μmol m^−2^·s^−1^)/8 h dark conditions. For growth rate analysis, a tip of leaf-like structure of gametophytes in WT and ∆*abi3* plants were cultured on BCD medium containing 0, 10, 20, and 50 μM of ABA for 20 days and 60 days; 7-day-old protonemata of WT and ∆*abi3* plants were cultured on BCD medium containing 0, 10, 20, and 50 μM ABA for 14 days. For microscope observation, 7-day-old protonemata of WT and *abi3* plants were cultured on BCDA medium containing 0 and 10 μM of ABA for 2 days and 7 days; 7-day-old protonemata of WT plants were cultured on BCDA medium containing 0 and 0.4 μM of IAA for 5 days, and 7-day-old protonemata of ∆*abi3* plants were cultured on normal BCDA medium for 5 days. For RNA-seq and qRT-PCR analyses, 7-day-old protonemata of WT, and *abi3* plants were cultured on BCDA medium containing 10 μM of ABA for 0 h, 2 h, and 4 h. All of the experiments were repeated at least three times.

### 4.2. Stereoscopic Microscope

Microscope Axiozoom V16 (Carl Zeiss AG, Oberkochen, Germany) was used to shoot the microscopic images. Black cardboard served as a background. The magnification was 112×. Images were exported from the ZEN lite software (Zeiss). All of the images were shot from at least three independent colonies. The stereoscopic microscope was used to measure the branches of each chloronemal cell and the ratio of caulonemal branches to total branches [23,39]. All of the data were taken for at least 40 colonies.

### 4.3. Measurement of Auxin Concentration by Enzyme-Linked Immunosorbent Assay (ELISA)

After grinding 200 mg of protonemata and gametophytes in liquid nitrogen, an extraction solution of 80% (*v*/*v*) methanol was added. The auxin of protonemata and gametophytes tissues was extracted at 4°C for 4 h. After centrifugation, the supernatants were measured with a plant auxin ELISA kit (Immuno-Biological Laboratories Inc., Minneapolis, MN, USA) according to the manufacturer’s instructions. Five biological replications were performed.

### 4.4. RNA Isolation and Sequencing

Total RNA was extracted from the protonemata of *P. patens* using RNeasy Plant Mini Kit (Qiagen, Valencia, CA, USA) and digested with TURBO DNA-free^TM^ Kit (Thermo Fisher Scientific Inc., Waltham, MA, USA) to remove DNA contamination. RNA integrity was evaluated using the Agilent 2100 Bioanalyzer (Agilent Technologies, Santa Clara, CA, USA). mRNA was enriched from total RNA by magnetic beads with an Oligo-dT tag, and double-stranded cDNA was synthesized using random-hexamer primer and reverse transcriptase (Superscript II, Invitrogen, CA, USA). After purification with magnetic beads, cDNA was connected with a tail of adenine and sequencing adapters, and then a cDNA library was constructed by PCR amplification. The resulting cDNA library was quantified with the Agilent 2100 Bioanalyzer (Agilent Technologies, Santa Clara, CA, USA). Subsequently, the cDNA library was sequenced on an Illumina HiSeq X-ten sequencer (Illumina, San Diego, CA, USA) at Oebiotech Co., Ltd. (Shanghai, China), and 150 paired-end base pair reads were generated.

### 4.5. RNA-Seq Data Preprocessing

The raw reads in the fastq format were first processed using an next generation sequencing (NGS) quality control (QC) Toolkit program (http://59.163.192.90:8080/ngsqctoolkit/). High-quality and clean reads were obtained by removing adapter sequences, low-quality reads, and uncertain bases (N). All of the downstream analyses were based on clean data of high quality determined by Q30. The sequencing reads were mapped to the complete genome of *P. patens* (https://phytozome.jgi.doe.gov/pz/portal.html#!info?alias=Org_Ppatens:PhytozomeV12:Ppatens_318_v3.fa.gz) and using TopHat software (http://ccb.jhu.edu/software/tophat/index.shtml). The fragments per kilobase of gene per million mapped reads and count values were calculated using eXpress (http://www.rna-seqblog.com/express-a-tool-for-quantification-of-rna-seq-data/).

### 4.6. DEGs and Functional Enrichment Analyses

The DEGs were identified using the DESeq (http://www.bioconductor.org/packages/release/bioc/html/DESeq.html) for six groups. FDR < 0.05 was used as the threshold of *p* value in multiple tests to determine the significance of differing expression of genes. Genes whose fold change ≥2 with a significance threshold of *p* < 0.05 were considered to be differentially expressed and were selected for further analysis. The DEGs were annotated against the *P. patens* database (https://phytozome.jgi.doe.gov/pz/portal.html#!info?alias=Org_Ppatens:PhytozomeV12:Phytozome:Ppatens_318_v3.3.gene.gff3.gz). GO enrichment analyses of DEGs were performed according to biological process ontologies (http://www.geneontology.org/) using BLAST software.

### 4.7. Quantitative Real-Time Reverse Transcription PCR (qRT-PCR) Analysis

Total RNA was extracted using an RNeasy Plant Mini Kit (Qiagen, Valencia, CA, USA), DNA was digested by a TURBO DNA-free^TM^ Kit (Thermo Fisher Scientific Inc., Waltham, MA, USA), and then RNA was reversed to cDNA with a PrimeScript Double Strand cDNA Synthesis Kit (Takara Bio, Dalian, China) according to the manufacturer’s instructions. The *P. patens* tubulin cDNA gene was used as a standard to normalize the content of cDNA. The qRT-PCR reactions were performed using gene-specific primers (Table S1) on an ABI7500 (Applied Biosystems, Foster City, CA, USA). A SYBR Premix Ex Taq (Perfect Real Time) kit was used for quantification of DEG sequences. The PCR reaction condition was as follows: 95 °C predenaturation for 10 min, 95 °C denaturation for 15 s and 60 °C for 50 s annealing (40 cycles), followed by a melting-curve step from 60 °C to 95 °C with a ramp rate of 2%. The efficiency of the primers was detected through calibration curves with a 1:5 dilution series and at least four points fitted in a linear regression with an R-square of over 0.99. The 2^–ΔΔCT^ method was used to analyze the data. The qRT-PCR data were normalized to the housekeeping gene: tubulin gene. All of the analysis included more than three biological replicates.

### 4.8. Statistical Analysis

All of the data were presented as the mean ± standard error (X ± SE). Statistical analysis was performed using a one-way analysis of variance (ANOVA) test with SPSS 18.0 software. *p* values of 0.05 or less were considered statistically significant. * indicates a significant difference compared to the control (0.01 < *p* < 0.05); ** indicates an extremely significant difference compared to the control (*p* < 0.01).

## 5. Conclusions

In conclusion, ABA and PpABI3 could regulate the vegetative development of the moss *P. patens*. Application of exogenous ABA suppressed the vegetative growth in both WT plants and ∆*abi3* mutants. The growth rate of ∆*abi3* mutants was faster than that of WT plants with or without ABA treatment. Therefore, PpABI3 is a negative regulator of the vegetative development in *P. patens*. ABA regulated vegetative development partly through PpABI3, and other regulators might be also involved in regulating vegetative development in the moss *P. patens*. Auxin concentrations of protonemata and gametophytes in ∆*abi3* mutants were significantly higher than that of WT plants. Exogenous IAA promoted the differentiation of WT plants from chloronema to caulonema and this phenotype was similar to ∆*abi3* mutants without IAA treatment. Auxin-related genes were identified in WT plants and ∆*abi3* mutants with or without ABA treatment, indicating that ABA and PpABI3 regulated vegetative development of *P. patens* through controlling the expression auxin synthesis, transport, and signaling pathways and that PpABI3 might be associated with cross-talk between ABA and auxin in the moss *P. patens*.

## Figures and Tables

**Table 1:** Differential expression of auxin-related genes; ∆*abi3* plants compared with WT plants.

Gene ID	Gene Symbol	WT-2 h vs. WT-0 h	∆*abi3*-2 h vs. ∆*abi3*-0 h	∆*abi3*-0 h vs. WT-0 h ∆*abi3*-2 h vs. WT-2 h ∆*abi3*-4 h vs. WT-4 h
		WT-4 h vs. WT-0 h	∆*abi3*-4 h vs. ∆*abi3*-0 h	
Pp3c11_11790	YUC6			U U U
Pp3c1_11500	YUC6			U U U
Pp3c13_21970	YUC8	D D	D D	U U U
Pp3c12_5490	AUX1	D D	D D	D D D
Pp3c15_640	AUX1	D D	D D	D D D
Pp3c19_12670	AUX1			D D D
Pp3c22_23050	AUX1	D D		D D D
Pp3c3_9870	AUX1	D D		D D D
Pp3c9_620	AUX1	D D	D D	D D D
Pp3c6_27190	AUX1	D D	D D	
Pp3c9_3590	AUX1	U U	U U	D D D
Pp3c14_8850	PIN4	D D	D D	D D D
Pp3c23_10200	PIN7	D D	D D	D D D
Pp3c24_2970	PIN4	D D	D D	D D D
Pp3c17_16450	ABCB19			U U U
Pp3c6_7260	ABCB19	D D	D D	U U U
Pp3c12_11230	ABCB4	D D	D D	U U U
Pp3c24_6610	IAA7	D D		U U U
Pp3c8_14720	IAA7			U U U
Pp3c4_13010	ARF16	D D	D D	U U U
Pp3c5_9420	ARF2	D D	D D	U U U
Pp3c1_14480	ARF6	D D	D D	U U U
Pp3c2_25890	ARF6		D D	U U U
Pp3c4_12970	ARF16	D D		U U U
Pp3c6_21370	ARF7		D D	
Pp3c6_26890	ARF16		D D	
Pp3c9_17560	ARF3	D D		
Pp3c9_21330	ARF6	D D	D D	
Pp3c13_4720	ARF6	D D	D D	
Pp3c14_16990	ARF6	D D	D D	
Pp3c15_21880	ARF3	D D		
Pp3c15_9710	ARF6	D D	D D	

U represents the upregulation of gene expression; D represents the downregulation of gene expression. Gene symbols were genes of *A. thaliana* which were searched by BLAST on the basis of the *P. patens* genes sequences according to the *Arabidopsis* database: https://www.arabidopsis.org/.

## Supplementary Materials

Supplemental Table S1 Abundance change of individual different expression genes involved in cell growth/division/differentiation. Supplemental Table S2 Abundance change of individual different expression genes involved in cell structure. Supplemental Table S3 Abundance change of individual different expression genes involved in signal transduction. Supplemental Table S4 Abundance change of individual different expression genes involved in response to light. Supplemental Table S5 Abundance change of individual different expression genes involved in transcription. Supplemental Table S6 Abundance change of individual different expression genes involved in ion transport. Supplemental Table S7 Abundance change of individual different expression genes involved in response to hormone. Supplemental Table S8 Primer pairs used in Quantitative Real-Time PCR.

## Abbreviations

ABA, abscisic acid; ABI, abscisic acid insensitive; ARF, auxin response factor; ATP, adenosine triphosphate; BBM, baby boom; cDNA, complementary DNA; DGE, differentially expressed genes; DNA, deoxyribonucleic acid; FDR, false discovery rate; IAA, indole-3-acetic acid; mRNA, messenger RNA; PP2C, clade a protein phosphatase type 2c; PYR/PYL/RCAR, pyrabactin resistance 1 (pyr1)/pyr1-like (pyl)/regulatory components of aba receptors; qRT-PCR, real-time polymerase chain reaction; RNA, ribonucleic acid; RNA-seq, RNA sequencing; SnRK2, sucrose nonfermenting 1-related protein kinase 2.
